# In vivo anti‐Salmonella properties of aqueous extract of prickly pear (*Opuntia ficus indica*) cladode, hepatological and toxicological evaluation

**DOI:** 10.1002/fsn3.4123

**Published:** 2024-03-25

**Authors:** Khansa Iftikhar, Farzana Siddique, Kashif Ameer, Muhammad Arshad, Sadia Kharal, Isam A. Mohamed Ahmed, Shakila Khalid

**Affiliations:** ^1^ Institute of Food Science and Nutrition University of Sargodha Sargodha Punjab Pakistan; ^2^ Department of Zoology University of Sargodha Sargodha Punjab Pakistan; ^3^ Department of Food Science and Nutrition, College of Food and Agricultural Sciences King Saud University Riyadh Saudi Arabia

**Keywords:** cefixime, cladode, diarrhea, erythropoietin, inflammation

## Abstract

The present study aimed to evaluate antidiarrheal potential of prickly pear cladode and its hepatoprotective role in different groups of diarrhea‐induced mice. Mice received cefixime (4 mg/kg of bw) and different concentrations of aqueous cladode extract (250 mg/kg of bw, 500 mg/kg of bw and 1000 mg/kg of bw). Feces *Salmonella typhi* ATCC 19430 was used to assess antidiarrheal potential and hematological, biochemical parameters, and histopathological analyses were carried out for 17 days. The results showed that administration of *Salmonella typhi* ATCC 19430 in mice produced liver injuries with histological damage, whereas 1000 mg/kg of bw cladode extract reduced the *Salmonella typhi* ATCC 19430 load of feces earlier as compared to the other groups during 17 days. Hematological parameters, like red blood cells (RBCs) (6.19 ± 1.85 × 10^6^/mm^3^) and hemoglobin (Hb) (10.06 ± 2.03 g/dL), of negative control group decreased, while white blood cells (WBCs) (13.46 × 10^6^/mm^3^) increased from reference values. In lipid profile, low‐density lipoprotein (LDL) (9.0 ± 2.41 mg/dL), high‐density lipoprotein (HDL) (6.07 ± 2.45 mg/dL) and total cholesterol (TC) (35.22 ± 8.29 mg/dL) of negative control group decreased, while triglycerides (TG) (168.35 ± 71.75 mg/dL) increased from reference values. Alanine transferase (ALT) (60.30 ± 20.33 IU/L), alkaline phosphatase (ALP) (359.9 ± 100.05 IU/L) and aspartate transferase (AST) (168.77 ± 66.61 IU/L) of negative control group increased from reference values. Urea (27.36 ± 10.54 mmol/L) and creatinine (35.29 ± 12.15 mmol/L) of the negative control group increased. Cefixime also ameliorated injuries due to the administration of *Salmonella typhi* ATCC 19430. Conclusively, these findings indicated that pure aqueous extract of cladode showed more promising results regarding antidiarrheal potential. Hence, cladode might be used in food and supplement formulations as a functional ingredient.

## Graphical abstract

This study was aimed to elucidate in vivo anti‐salmonella properties of aqueous extract of prickly pear (*Opuntia ficus indica*) cladode. The bacterial load of feces was calculated a day before the infection and was continually calculated during 4 days after the infection. Histology of the liver continued to play an important role in modern hepatological practice.

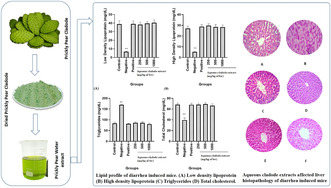

## 1 INTRODUCTION

Natural products have a particular chemical structure due to which these products exhibit several biological activities and nutraceutical properties. Different bioactive compounds, such as tannins, alkaloids, saponins, flavonoids, steroids and terpenoids, possess antidiarrheal activity (Alemayehu et al., 2022). Prickly pear (*Opuntia ficus indica*) has been reported to be originated in Mexico. Prickly pear is produced in several countries around the globe, such as South Africa, Italy, Spain and multiple countries in South America (especially Argentina, Chile and Brazil). Prickly pear fruit pulp, peel, cladode, and seeds have therapeutic properties and are non‐toxic for human utilization. Their pharmacological activity is dependent on different factors, such as components used, extraction method type, as well as phytochemical compounds present in extract (Eman et al., 2022). Feugang et al. (2020) determined that the prickly pear fruit and cladodes extracts might be employed to prepare food supplements and nutraceuticals.

Prickly pear fruit has many health benefits: hypolipidemic and hypoglycemic activities. It comprises rich amounts of ascorbic acid, vitamin E, fiber, carotenoids and antioxidant components (flavonoids, phenols, betaxanthin and betacyanin) and amino acids (glutamic acid, aspartic acid, arginine and glycine) (Osorio et al., 2011; Paiz et al., 2010). Scientific studies have demonstrated that the prickly pear contains different bioactive compounds, such as water‐soluble pigments (betelains), flavonoids and phenolic acids (piscidic acid, quercetin, etc.), vitamin B complex (B_1_, B_2_, B_3_ and B_6_), and soluble and insoluble dietary fibers. These compounds have significant antimicrobial activity against different microbes including *Escherichia coli*, *Helicobacter pylori*, *Staphylococcus aureus* and *Salmonella typhi*.

According to the World Health Organization (WHO), diarrheal diseases account for an estimated 1.5 million deaths per year globally. The prevalence of diarrhea varies by region, with the highest burden found in sub‐Saharan Africa and South Asia (Schiller et al., 2017). Children who died due to diarrhea are even more than those who died due to AIDS, malaria, measles, injuries and all other post‐neonatal conditions combined. Different bacteria like *Salmonella typhi* and *Candida albicans* are the main agents that can be responsible for increasing incidence of diarrhea. Pathogenic bacteria are responsible for increasing mortality rate in humans (Alam et al., 2021). The mechanism by which *Salmonella* damages the liver involves several steps, such as *Salmonella* initially invades the intestinal epithelial cells after ingestion of contaminated food or water. The bacteria exhibit adherence and penetrate the intestinal lining. Then, once inside the host, *Salmonella* can enter the bloodstream and disseminate throughout the body, reaching distant organs, including the liver. *Salmonella* can actively migrate to the liver through the bloodstream, where it infiltrates liver tissue. The liver is a major organ involved in detoxification, metabolism and immune response. *Salmonella* infection triggers an immune response, leading to the activation of immune cells (macrophages) in infected liver tissue. In some cases, *Salmonella* infection can lead to the formation of granulomas in the liver. Granulomas are organized clusters of immune cells that attempt to contain the infection (Abdulgafor et al., 2018; Rishi et al., 2009).

*Salmonella typhi* belongs to the family *Enterobacteriaceae* that is mostly present in the intestines of humans and animals. It is widely present in nature and is a causative agent of food poisoning and illness in humans and animals (Lee et al., 2013). The exact time for treatment of *Salmonella gastroenteritis* has not been defined but according to most doctors, it can be cured within 5 to 7 days. Previously published research report by Wang et al. (2019) has reported on about *Kalimeris indica* ethanolic extract in vivo hepatoprotective effects. The authors have concluded that the extract led to improvement in liver injury in mice and decreasing tendencies in hepatic malondialdehyde (MDA) content and total nitric oxide synthase (tNOS) levels. Moreover, *Artemisia annua* L. extract has also been investigated regarding recovery of acute liver failure by Park et al. (2020). In an animal model of acute liver failure, oral administration of *A. annua* L. extract reduced the levels of liver enzymes, such as AST and ALT, indicating protection against liver damage. Similarly, Kim et al. (2020) have reported on garlic fermented by lactic acid bacteria (LAB) which exhibited hepatoprotective effects. Fermented garlic extract showed antioxidant activity and increased levels of S‐allyl‐l‐cysteine, total thiosulfinate content, total flavonoid content, and ferric‐reducing antioxidant power. Samadi‐Noshahr et al. (2021) have evaluated the in vivo hepatoprotective effects of fennel seed extract. Fennel seed extract and its active compound, such as trans‐anethole, have been shown to be effective in preventing streptozotocin‐induced liver injury in rats. Both fennel and trans‐anethole were found to decrease blood glucose levels, reduce liver enzyme activity and improve lipid profile in diabetic rats. However, there is no published report available to the best of our knowledge on investigation of in vivo anti‐salmonella properties of aqueous extract of prickly pear cladod and hepatological and toxicological effects. Therefore, the goal of the current study is to investigate the protective effect of cladode against *Salmonella typhi* in mice and clarify that *Opuntia* cladode serves as a possible treatment for diarrhea. Histopathological analysis was also performed to evaluate the effect of prickly pear cladode extract on the liver.

## 2 MATERIALS AND METHODS

### 2.1 Plant material

Prickly pear fruits (Meyer variety) along with cladodes were procured from the Talagang District, Pakistan. Experiments were carried out in the laboratory of the Institute of Food Science and Nutrition, University of Sargodha, Sargodha, Pakistan. Fruits and cladodes were separately washed to remove dirty material and were air‐dried and stored in air‐tight glass containers at refrigerated temperature ranged 4–6°C until further analysis.

### 2.2 Test bacterium and culture medium

*Salmonella typhi* ATCC 19430 was used in this study and provided by the Institute of Microbiology, Faculty of Veterinary Science, University of Agriculture, Faisalabad. Agar slant was used to maintain the bacterial strain at 4°C and sub‐cultured on a media plate for 24 h before antimicrobial test. For bacterial counts and identification, *Salmonella*‐*Shigella* agar was used in mice.

### 2.3 Experimental animals

Male mice (weighing 38–40 g) were used in the study. They were bred at the animal house of University of Agriculture, Faisalabad at specific temperature (23 ± 2°C). The study was approved by the Institutional Review Board (IRB) of University of Sargodha, and the number of the IRB approval of the research study is IRB No. UOS‐1 (104)/dean/agri/2120:11‐11‐2016.

### 2.4 Chemicals for antimicrobial assay

Cefixime and cyclophosphamid procured from clinics were used as the reference antibiotic and immuno‐suppressor, respectively.

### 2.5 Preparation of aqueous prickly pear cladode extracts

Fresh cladodes extraction was carried out according to the method described by Benayad et al. (2014) with slight modifications. Cladodes (20 g) were ground with water, put in a separate conical flask and placed in a shaking incubator (Shing Saeng Skir‐601 L, Japan) at 22–25°C at 40 rpm for 24 h. Macerated extracts were then filtered using Whatman No.1 filter paper, and water was evaporated at 45°C and 60 rpm in a rotary evaporator (Heidolph Laborota 4001, Japan).

### 2.6 Bio‐assay studies

#### 2.6.1 Induction of diarrhea in mice

Mice were divided into six groups and each group contained 10 mice. Different treatments, such as *T*_0_, *T*_1_, *T*_2_, *T*_3_, *T*_4_ and *T*_5_, were represented by control, negative control, positive control (4 mg/kg of bw), *T*_1_ (250 mg/kg of bw), *T*_2_ (500 mg/kg of bw) and *T*_3_ (1000 mg/kg of bw), respectively. Mice were kept in cages for 1 week before the experiment. Before administration of strain, mice were fasted overnight and given 1 mL of saline solution containing 1.5 × 10^8^ CFU of *Salmonella typhi* ATCC 19430 to all groups except the control group. Strain was given to the negative control group. Strain and cefixime (4 mg/kg bw) were given to the positive control group. Strain and cladode extract were given to treatment groups 1, 2 and 3. The bacterial load of feces was calculated a day before the infection and was continually calculated for period of 4 days after the infection. After giving bacterial suspension, collection of fecal samples was performed every day, and the number of bacteria/g of feces was calculated. 1 g of feces was dissolved in 2 mL saline, and then 100 μL of the fecal mixture was serially diluted by using saline distilled water and plated on agar plates. Then incubation was carried out at 37°C, and colonies were counted (Donald et al., 2015).

#### 2.6.2 Blood collection and serum sample preparation

Blood was collected, and serum was prepared by using the method of Donald et al. (2015). After the experiment, mice were anesthetized (chloroform) before dissection. Blood was taken by cardiac puncture, and two test tubes were used for the collection of blood. Ethylene diamine tetraacetic acid (EDTA) was present in one tube, and no anticoagulant was present in the other tube. The anticoagulant‐containing tube was used for hematological parameters, whereas blood poured into the tube having no anticoagulant was used for serum sample preparation. To prepare serum, clotting of blood was carried out by putting it at 37°C for 1 h, and then refrigeration was performed for another 1 h. Resultantly, the supernatant was obtained. Centrifugation of supernatant was carried out at 1058 rpm for 10 min, and then the clear serum was obtained and stored at −30°C for further analysis.

### 2.7 Hematological assays

White blood cells (WBCs), red blood cells (RBCs) and hemoglobin (Hb) were determined by using an automated blood analyzer (QBC Autoread Plus, UK) (Donald et al., 2015).

### 2.8 Biochemical assays

The prepared serum was used to determine total cholesterol (TC) and serum triglycerides (TG), which were determined by the method discussed by Notio et al. (2003). High‐density lipoprotein (HDL) cholesterol was estimated. Low‐density lipoprotein (LDL) cholesterol was calculated by Equation 1 reported by Friedewald et al. (1972) given below:

(1) $$\mathrm{LDL}=\mathrm{TC}-\mathrm{HDL}-\left[\;\frac{\mathrm{TG}}{5}\;\right]$$

Liver enzymes (ALT, ALP and AST) were estimated by the methodology discussed by Sher and Hung (2009). Urea and creatinine were determined by the method described by Pierre et al. (2017).

### 2.9 Histopathological analysis

For histopathological analysis, cross‐sections of liver were prepared and examination was done through methods described by Donald et al. (2015). For that purpose, animals were sacrificed, and then liver and stomach sections were placed in 10% formaldehyde and washed with xylene. These sections were enclosed in wax made up of paraffin and with the help of a rotary microtome and were suctioned up to 5 μm thickness. After this, hematoxylin and eosin stainings were performed. Then, the light microscope was used to analyze the sections of the liver and stomach, and microscopic camera was used to take photograph (Donald et al., 2015).

### 2.10 Statistical analysis

Experimental data were expressed as means ± SD (standard deviation). Differences between groups were evaluated by one‐way analysis of variance (ANOVA) with Tukey's multiple comparison test. Statistical analysis was performed using GraphPad Prism software (GraphPad Prism 8.3). Tukey's test was used to statistically evaluate the data (Statistics 8.1). The *p‐*value of < .01 was considered to be statistically significant.

### 2.11 Ethics

This work was carried out for the welfare of animals as recommended by NENT (2019).

## 3 RESULTS AND DISCUSSION

### 3.1 In vivo antidiarrheal activity of aqueous extract of *Opuntia ficus indica* cladode

**FIGURE 1:**
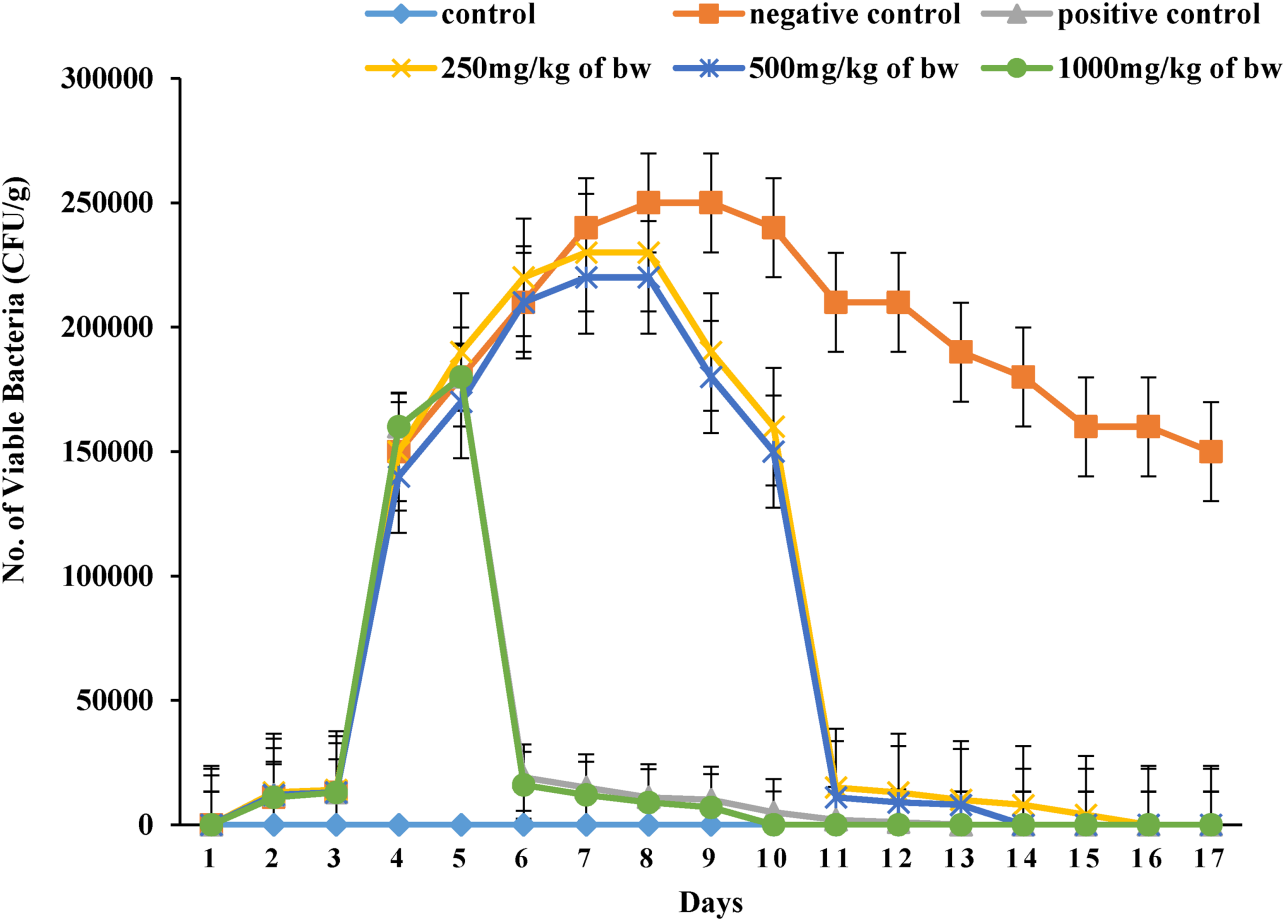
Effect of various cladode aqueous extract treatments on fecal shedding (CFU/g) of *Salmonella typhi* in mice.

*Salmonella typhi* obtained from feces increased during the first day of the infection. The intake of prickly pear cladode extract decreased in the *Salmonella typhi* obtained from feces as shown in Figure 1. 250 mg/kg of bw and 500 mg/kg of bw of the extract given to the animals did not result in decreasing tendency of *Salmonella typhi* in feces between the fifth and eighth days of the experiment. The same observation was obtained for those animals given standard antibiotics and 500 mg/kg of bw cladode extract on day 5 of the treatment period. The *Salmonella typhi* also decreased in the feces of infected and untreated control animals, but this only occurred after completion of 4 to 5 days in case of treated animals. The current findings obtained from the biological study of diarrhea showed that the growth of *Salmonella typhi* was inhibited after administration of an aqueous extract of cactus pear cladode. After starting the experiment, the decrement of microbial load in infected animals occurred due to the mutual effect of the immune system and cladode extract. Whereas a decrease in low microbial load was also noted in animals of the negative control group, this only happened in *T*_1_ after 5 to 6 days as compared to those of positive control and other treatment groups. These findings of the current research are in concordance with the work of earlier scientists (Donald et al., 2015) who stated that the reduction of microbial load in infected and treated animals was dependent on the dose, and their microbial growth was completely removed within 14 to 17 days of treatment. Previous research by Lutterodt et al. (1999) indicated that flavonoids present in the extract were responsible for antidiarrheal effect of the extract. The results of phytochemicals analysis (data not shown) indicated that antidiarrheal activity of prickly pear cladode extract against *Salmonella typhi* might be ascribed to the effect of these compounds (Kouitcheu et al., 2011). Some other researchers proposed that flavonoids are effective antioxidants and showed strong antidiarrheal activities (Del & Gutiérrez, 2008). *Salmonella enterica*, including *Salmonella typhi* and *Salmonella paratyphi*, cause severe diseases, such as typhoid and paratyphoid fevers, which are major global public health problems. Antibiotics are commonly used to treat salmonellosis, but the increasing resistance of *Salmonella typhi* to these drugs necessitates the search for new antityphoid agents. Similar to results of the current study, Donald et al. (2015) have reported the findings about assessment of in vivo anti‐salmonella activity of aqueous extract of prostrate spurge (*Euphorbia prostrata*) against *Salmonella typhimurium*, a strain that induces a systemic infection in rats similar to typhoid fever in humans – as well as its toxicological effects were evaluated. Prostrate spurge, a plant abundantly found in India and Africa, has been traditionally used for various medicinal purposes including digestive system disorders. Previous studies have shown that prostrate spurge extracts have antimicrobial activity against *Salmonella typhi* and other *Salmonella* serotypes. The safety of the extract was also assessed through subacute toxicological study. Authors have reported that the aqueous extract of *E. prostrata* showed significant anti‐salmonella activity in a rat model, reducing the number of viable *Salmonella typhimurium* in feces and effectively treated salmonellosis. The extract was found to be non‐toxic at certain doses, with no significant (*p* > .05) changes in food and water consumption patterns, and an increase in weight gain was observed during the treatment period. However, at higher doses, the extract could induce liver damage and inflammation, as indicated by elevated serum transaminases levels and histopathological analysis. Side effects were also observed in case of kidneys, as shown by changes in serum, urinary creatinine and urinary protein levels. The hematological status of the rats after 14 days of extract administration showed no significant differences (*p* > .05) compared to that of control groups, except for anemia observed in infected and untreated female rats, possibly due to the effects of *Salmonella* infection. In another study carried out by Olatoye and Arueya (2018), whereby authors have evaluated the safety of aerial yam and *Treculia Africana*, Murine model involving 30 male albino rats was employed for time interval of 28 days. The blood samples of rats were then subjected to the hematological and biochemical assays followed by histological analysis. Authors have conclusively reported that hematological and biochemical parameters did not differ significantly (*p* > .05) among all treatments as compared to those of control. The novel snack food exhibited the immune‐boosting potential with gradual rise in the monocytes. When kidney and liver of snack‐fed rats were observed, it was found that the rats fed with the snacks did not show any significant pathological changes. No signs of toxicity were observed in murine within the investigated time period of 28 days. It was evident from the findings that aerial yam and *Treculia Africana* snacks could be utilized as the useful immune adjuvant with health‐beneficial properties. In a report by Rahmani et al. (2023), rutin is categorized as the flavonoid originated from the plant species. It exhibits anti‐inflammatory, antiapoptotic and antioxidant properties. In a report published by the whereby authors have reviewed potential hepatoprotective, renoprotective and cardioprotective effects of Rutin. Rutin antioxidant potential was ascribed to the increasing tendency of different enzymes, such as GST, GGT, CAT, GPx, SOD and GR which resulted in activation of Nrf2/HO‐ 1 pathway. Rutin also exerted its antiapoptotic effects owing to inhibition of free radicals as well as elevation of Bcl‐2 protein. Moreover, rutin also exhibited therapeutic effects against several antioxidants (carbon tetrachloride, thioacetamide, cadmium, mercuric chloride and tert‐butyl hydroperoxide).

### 3.2 Effect of aqueous cladode extract on hematological parameters of diarrhea‐induced mice

The results regarding the influence of aqueous cladode extract with respect to treatments and days on hematological parameters of diarrhea‐induced mice are given in Table 1. It is evident from the results that RBCs and Hb of mice of the negative control group (*T*_1_) significantly (*p* < .01) differed from the standard values, while the WBCs of mice of *T*_1_ significantly (*p* < .01) increased when compared to the standard values. It was also observed that after 16 days of the experiment, RBCs and Hb decreased while WBCs increased from the reference values. Statistically, hematological parameter values of *T*_0_, *T*_2_, *T*_3_, *T*_4_ and *T*_5_ remained within the standard ranges and were not influenced by the days and treatment variables. In case of infected mice in present study, low Hb levels might be due to different factors, such as blocking the iron release, reduction in the intestinal absorption of iron and inhibition or inappropriate production of erythropoietin. This observation was confirmed by Levy et al. (2005). Reduction in RBCs was due to the effects of inflammation‐causing cytokines on erythroidprogenitor cells. This observation was supported by Keast and Fraser (2004). Increase in WBCs in infected mice was due to inflammatory mediators. These results were in agreement with the finding of Campos et al. (2010).

**TABLE 1:** Effect of aqueous cladode extract on hematological parameters of diarrhea‐induced mice.

Groups	RBCs (×10^6^/mm^3^)	WBCs (×10^6^/mm^3^)	Hb (g/dL)
Control	8.49 ± 0.32^a^	6.68 ± 0.37^b^	12.68 ± 0.33^a^
Negative control	3.91 ± 0.12^b^	20.47 ± 0.64^a^	7.58 ± 0.21^b^
Positive control	8.61 ± 0.33^a^	6.63 ± 0.35^b^	12.61 ± 0.67^a^
250 mg/kg of bw	8.73 ± 0.43^a^	6.46 ± 0.33^b^	12.81 ± 0.66^a^
500 mg/kg of bw	8.38 ± 0.21^a^	6.31 ± 0.31^b^	12.61 ± 0.57^a^
1000 mg/kg of bw	8.49 ± 0.32^a^	6.58 ± 0.20^b^	12.71 ± 0.66^a^

*Note*: Control: non‐infected and untreated, Negative control: infected and untreated, Positive control: infected and treated with drug; Results are mean ± standard deviation of three replicate determinations; Mean values presenting different small letters in rows are significantly different from each other.

Another study was conducted by Legba et al. (2020), and in vivo anti‐salmonella activities of *Uvaria chamae*, *Lantana camara* and *Phyllantus amarus* (medicinal plants originated from Benin, West Africa) were assessed for their effects to treat salmonellosis. Authors have concluded that antibiotic resistance in *Salmonella* strains has become a global health concern, leading to the exploration of medicinal plants as an alternative treatment option. The findings suggest that the aqueous extract of *Uvaria chamae* shows promising anti‐Salmonella activity and could be developed as an improved traditional medicine for the management of salmonellosis. In vitro tests showed that the aqueous extract of *Uvaria chamae* leaves exhibited the best anti‐Salmonella activity. The ethanolic extract of *Phyllantus amarus* leaves, and both ethanolic and aqueous extracts of *Lantana camara* (leaves) also showed activity against *Salmonella typhimurium* ATCC 14028. In vivo experiments using chicks infected with *Salmonella typhimurium* ATCC 14028 showed that after 7 days of treatment, the aqueous extract of *Uvaria chamae* at concentrations of 100, 200 and 400 mg/L resulted in reductions of bacterial load by 85, 52.38 and 98%, respectively. Colistin completely eliminated the bacterial load. The aqueous extract of *Uvaria chamae* was also in vitro and in vivo activities against multi‐resistant strains of *Salmonella enterica*, making it a potential candidate for the development of traditional medicine for salmonellosis management (Legba et al., 2020). In a published report by Panritdum et al. (2024), authors investigated the potential impact of chronic liver diseases, such as fatty liver disease and hepatitis, on liver cells, with a focus on the development of fibrosis, chronic inflammation and cirrhosis. This study explored the relationship between these conditions and the increased risk of hepatocellular carcinoma, which is the leading cause of primary liver cancer. The study specifically examined the protective effects of *Cleistocalyx nervosum* fruit extract (CNPE) and cyanidin‐3‐glucoside (C3G) on liver cell damage and oxidative stress caused by hydrogen peroxide (H_2_O_2_).

### 3.3 Effect of aqueous cladode extract on lipid profile of diarrhea‐induced mice

**FIGURE 2:**
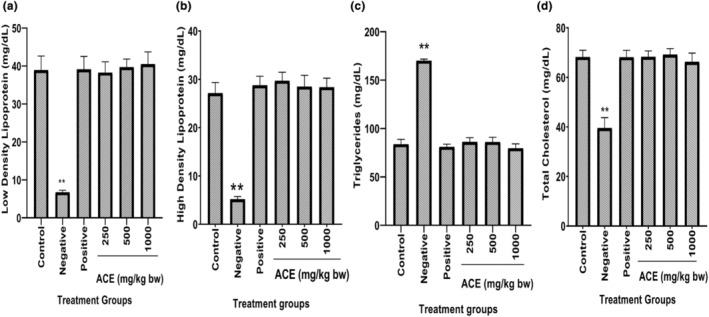
Effect of aqueous cladode extract on lipid profile of diarrhea‐induced mice. (a) Low‐density lipoprotein, (b) High‐density lipoprotein, (c) Triglycerides, (d) Total cholesterol. Control (non‐infected and untreated), Negative (infected and untreated), Positive (infected and treated with drug), 250 mg/kg of bw, 500 mg/kg of bw, 1000 mg/kg of bw (concentration of cladode aqueous extract). Data are expressed as the mean ± SDM. **p < .01, compared to the diarrhea control group. *, significant (*p* < .05), **, highly significant (*p* < .01).

The results of the effect of prickly pear cladode aqueous extract on the lipid profile after 17 days of treatment are shown in Figure 2. It appeared from results that there were significant (*p* < .01) changes in LDL, HDL, TC and TG of infected and untreated (negative control group) mice compared to those of control group. The LDL, HDL and TC showed significant (*p* < .01) decreases, while TG showed a significant (*p* < .01) increment in mice of the negative control group. All other groups showed non‐significant (*p* > .05) behavior compared to that of control group. Decreased levels of HDL from standard values might be attributed to a decrease in the synthesis of cholesterol, which has played an important role in the structure of these lipoproteins due to liver damage. This approach has been observed by Razavi et al. (2012). Serum triglyceride level was increased due to the activation of cytokines. Some other researchers found that cytokines were active due to lipopolysaccharide and endotoxins present in the cell wall structure of *Salmonella* spp. (Albayrak & Kabu, 2016; Feingold et al., 1995; Ly et al., 1995). Decreased LDL level in the present work might be due to an increase in triglyceride levels in liver. Elevation of TG and lowering of LDL, HDL and TC were also observed by Bozukluhan et al. (2017) who reported that HDL level has been increased during inflammation.

### 3.4 Effect of aqueous cladode extract on renal function tests (RFTs) of diarrhea‐induced mice

**FIGURE 3:**
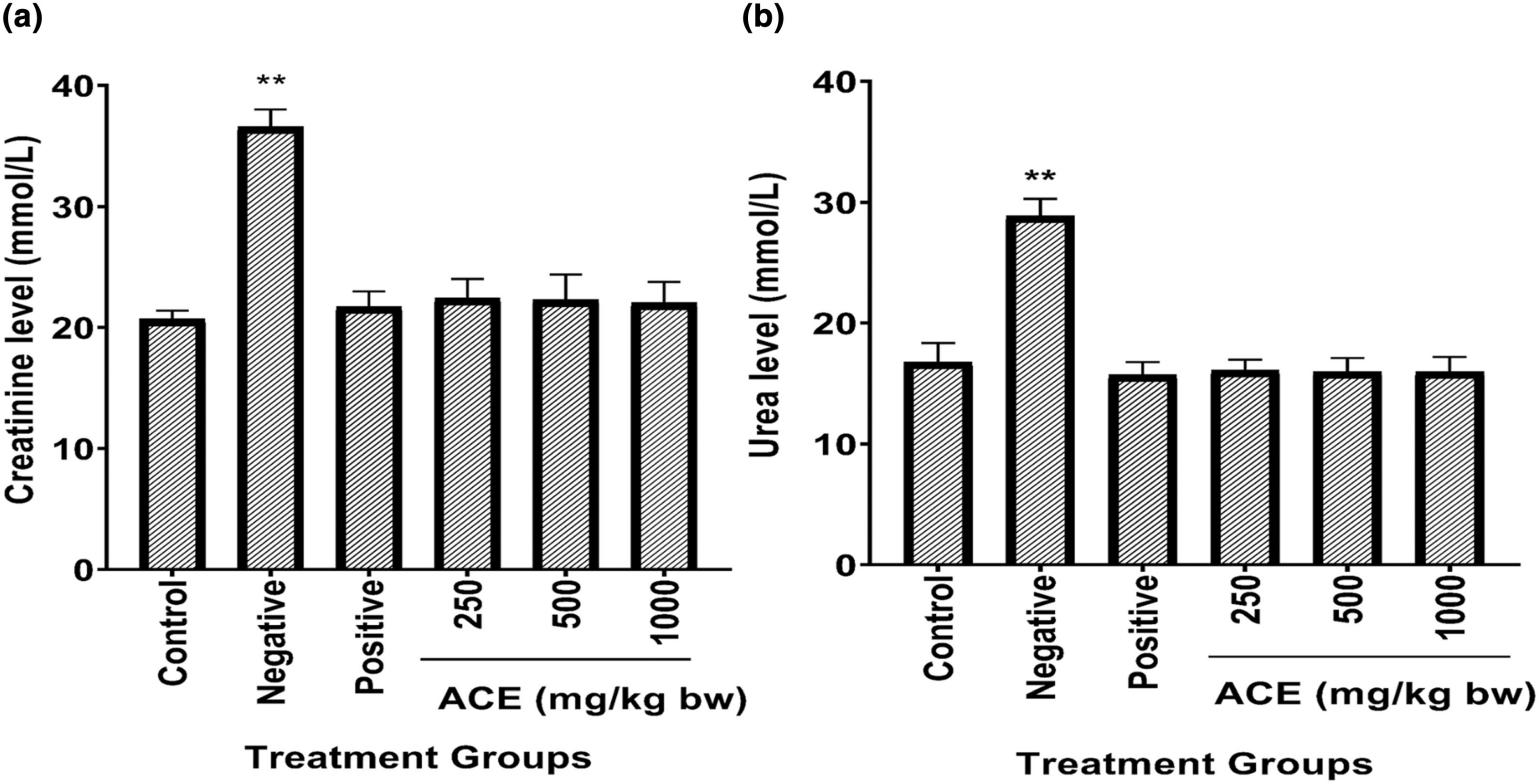
Effect of aqueous cladode extract on renal function tests (RFTs) of diarrhea‐induced mice. (a) Creatinine, (b) Urea. Control (non‐infected and untreated), Negative (infected and untreated), Positive (infected and treated with drug), 250 mg/kg of bw, 500 mg/kg of bw, 1000 mg/kg of bw (concentration of cladode aqueous extract). Data are expressed as the mean ± SDM. ** *p* < .01, compared to the diarrhea control group. *, significant; **, highly significant.

In general, there were significant changes in urea and creatinine levels of infected and untreated compared to the control group, while non‐significant (*p* > .05) changes were observed in urea and creatinine level of mice of other groups (Figure 3). In the present study, increase in serum creatinine and urea levels was according to the findings of earlier researchers (Baser & Civelek, 2013; Merhan et al., 2016). In another report by Rasool et al. (2023), authors have conducted research on the medicinal plants, such as *Argyrolobium roseum* (Camb.) Jaub & Spach (*Papilionaceae*), and evaluated the pharmacological effects of ethanolic and aqueous extracts of these medicinal plants on trace minerals like lead‐induced oxidative stress on vital body organs. The concentrations of different elements were measured by means of atomic absorption spectrophotometer in conjunction with DPPH and FRAP assays. After ingestion of aqueous and ethanolic extracts, the murine kidney and liver showed significantly reduced Pb levels in homogenates, especially in rats fed with 600 mg/kg aqueous extracts. It was evident from the results that aqueous extracts of *Argyrolobium roseum* (Camb.) Jaub & Spach exhibited the significantly less oxidative stress which led to reduced hepatic and renal damage via improvement of antioxidant compounds in treatment group of murine as compared to that of control.

### 3.5 Effect of aqueous cladode extract on liver function tests (LFTs) of diarrhea‐induced mice

**FIGURE 4:**
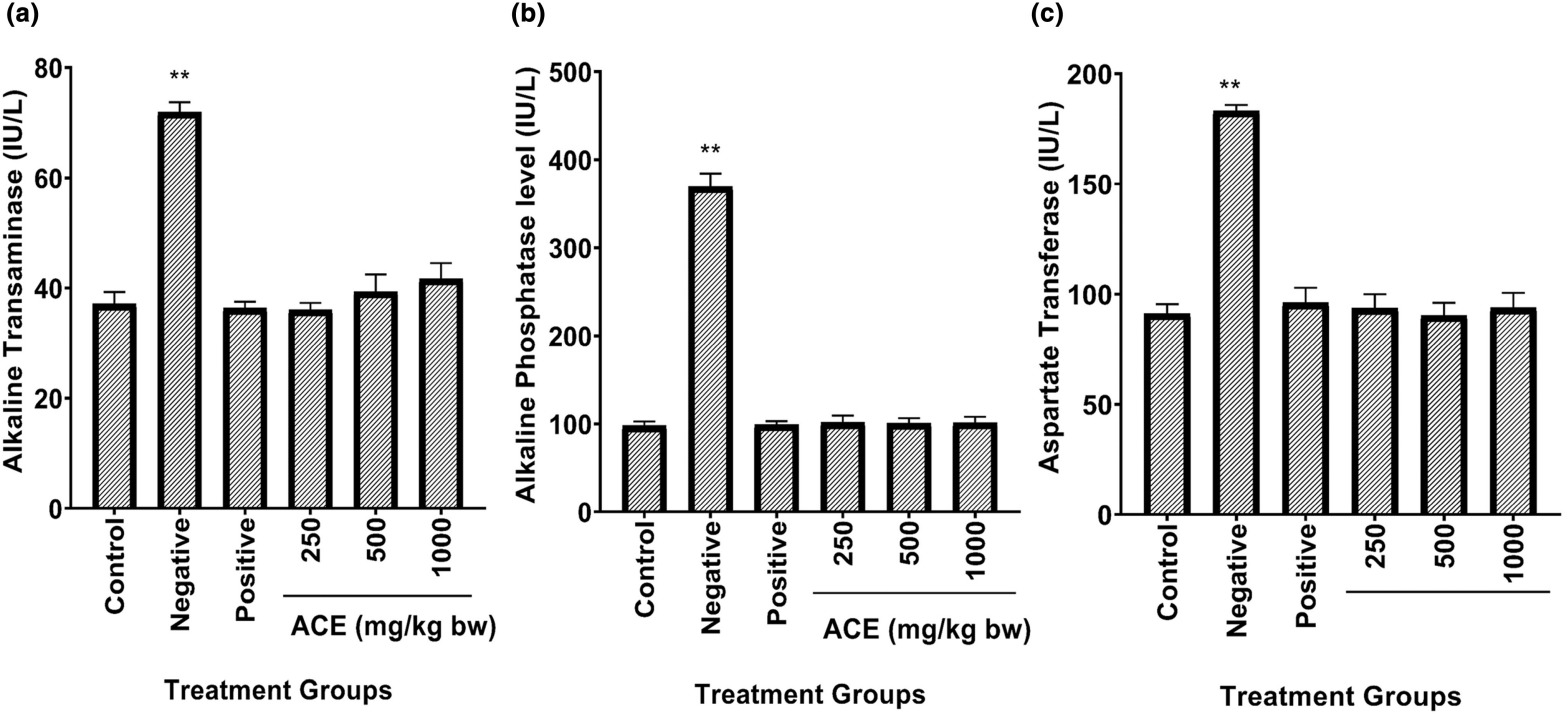
Effect of aqueous cladode extract on liver function tests (LFTs) of diarrhea‐induced mice. (a) Alkaline transaminase, (b) Alkaline phosphatase, (c) Asparate transaminase. Control (non‐infected and untreated), Negative (infected and untreated), Positive (infected and treated with drug), 250 mg/kg of bw, 500 mg/kg of bw, 1000 mg/kg of bw (concentration of cladode aqueous extract). Data are expressed as the mean ± SDM. **p < .01, compared to the diarrhea control group. *, significant; **, highly significant.

Figure 4 summarized the observations of aqueous extract effects of cladode on liver enzymes (ALT, AST and ALP) after 17 days of treatment. Levels of these enzymes were significantly (*p* < .01) increased in infected and untreated mice compared to the control group, while there were no significant (*p* > .05) changes in mice of other groups. Increased levels of AST, ALT and ALP in this study were in accordance with the findings of Baser and Civelek (2013) and Merhan et al. (2016). Liver enzymes (ALP, ALT and AST) were accumulated inside the cells of the liver under normal conditions. However, when the destruction of liver cells occurred, then these enzymes were moved into the bloodstream. As a result, the concentrations of these enzymes increased in blood (Nesreen et al., 2021) as observed in the current research. In a study by Omage et al. (2019), authors have antioxidant properties and phytochemicals of powder, ethanolic and aqueous extracts of ripe *Dennettia tripetala* fruits. Histological effects of these fruits were evaluated on healthy rats by using dose of 1000 mg/kg bw for period of 28 days. The extracts did not cause any significant change in serum protein profile, liver marker enzymes, creatinine and urea. The findings have suggested that *D. tripetala* fruits exhibit ethnomedicinal uses. The fruit extracts led to minimal centriole congestion on liver and did not cause any toxicity to vital organs, such as heart, kidney and liver. It was indicative from results that fruits of *D. tripetala* may be utilized as source of important phytochemicals in conjunction with medicinal properties.

### 3.6 Effect of aqueous cladode extract on liver histopathology of diarrhea‐induced mice

**FIGURE 5:**
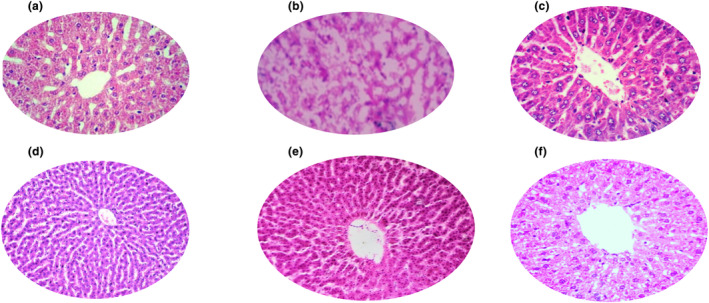
Effect of various doses (mg/kg bw) of aqueous cladode extracts on liver histopathology of diarrhea‐induced mice. (a) control (*T*_0_). (b) negative control group (*T*_1_) (infected and untreated). (c) positive control (*T*_2_) (infected and treated with drug). (d) treatment group 1 (*T*_3_) 250 mg/kg of bw of aqueous extract of cladode. (e) treatment group 2 (*T*_4_) 500 mg/kg of bw of aqueous extract of cladode. (f) treatment group 3 (*T*_5_) 1000 mg/kg of bw of aqueous extract of cladode.

Histological results of the liver sections are shown in Figure 5. The animals of positive control group at dose of 4 mg/kg of bw cefixime showed normal hepatic cells with veins similar to that of the control group as indicated in Figure 5c. The liver sections of negative control mice showed some disturbances in hepatic cells like vacuolization, and congested central vein as shown in Figure 5b. The liver sections of the animals treated with doses of 250 mg/kg of bw, 500 mg/kg of bw and 1000 mg/kg of bw of aqueous extract of *Opuntia* cladode showed clear nucleus, visible central vein and well‐preserved cytoplasm as indicated in Figure 5d–f. The hepatocellular damage was completely treated in all groups, and no histological alterations were observed except in case of negative control group.

In this study, cladode extract showed a protective effect on a chronic liver injury that might be due to the fact that cladode contained different bioactive compounds and exhibited high antioxidant activity as observed in the current study (data not shown). The exact mechanism of hepatoprotective effect has yet to be elucidated and is not exactly known. Previous researchers reported that lemon flavonoids exhibited hepatoprotective effect on carbon tetrachloride‐induced liver infection and the process of the protective effect was linked to the antioxidant capacity (Minato et al., 2003). The observations of current research are in concordance with the findings of previous workers (Anouar et al., 2017; Donald et al., 2015) and were also confirmed by antioxidant activity (data not shown). Leaves were important part of the medicinal plant, and intake through mouth was the only reported route. In other reports published by Dey and De (2012) and Nguyen et al. (2019), the most used medicinal plants for treatment of typhoid fever and gastrointestinal disorders were species from the *Cactaceae* family. Different published reports have revealed that leaves of medicinal plant could be employed for prevention of gastrointestinal disorders (Chegaing et al., 2020; Dey & De, 2012; Nguyen et al., 2019). Another study was carried out by Ozgun‐Acar et al. (2022). The research paper examined the biochemical, pharmacological, and toxicological attributes of *Capparis ovata* water extract (COWE). The study evaluates the mineral and fatty acid content, radical‐scavenging capacity, and anti‐inflammatory properties of COWE. The whole‐genome transcriptomic profiling reveals that COWE modulates immune responses, upregulates natural killer cell activation, and affects B‐cell proliferation and differentiation. Toxicological analysis indicates that COWE at or lower doses of 500 mg/kg/day does not cause adverse effects. COWE is found to be a rich source of nutrients with high antioxidant activity and anti‐inflammatory properties, making it a potential adjunct therapy for inflammatory diseases. The study also explores the potential toxicological effects of COWE, indicating that 250 and 500 mg/kg/day doses of *C. ovata* can be considered safe, while cautious consideration is advised for doses at or above 750 mg/kg/day.

The overall results of the recent research provided basis for the possible utilization of the aqueous extract of *Opuntia ficus indica* cladode diarrhea treatment. Results obtained in the present research revealed that the cladode extract is toxin free. These results showed that cladode can be used in the prevention of diarrhea. Antidiarrheal activity showed that the extract dose obtained from traditional healers may be considered relatively safe.

## 4 CONCLUSIONS

The findings of the present study provided convincing evidence that aqueous extract of *Opuntia ficus indica* cladode possessed remarkable antidiarrheal activity. Antidiarrheal effect was statistically significant (*p* < .05) at concentration of 1000 mg/kg dose. Moreover, the in vivo antibacterial activity revealed that the dose of extract obtained from traditional healer may be considered relatively safe. The practice of using conventional medicinal plants played a fundamental role in basic health needs in developing countries to fight with the multi‐drug‐resistant bacteria. In the present study, *Opuntia* cladode showed significantly (*p* < .05) higher antidiarrheal activity with the presence of various phytochemical constituents in it. Further studies of quantification are needed to bring out the therapeutic value of *Opuntia ficus indica* against the gut pathogens. This research provided the scientific basis for the utilization of *Opuntia ficus indica*. *Opuntia* cladode can be used for the treatment of diarrhea and also demonstrated that hematology and biochemical parameters may be considered valuable prognostic indicators to determine the effect of inflammation on diarrheic mice by using these parameters, the probability of correct prognosis can be improved substantially. Histology of the liver continued to play an important role in modern hepatological practice.

## AUTHOR CONTRIBUTIONS

**Khansa Iftikhar:** Conceptualization (equal); investigation (equal); methodology (equal). **Farzana Siddique:** Conceptualization (equal); investigation (equal); methodology (equal); supervision (equal); visualization (equal). **Kashif Ameer:** Conceptualization (equal); methodology (equal); validation (equal); visualization (equal); writing – review and editing (equal). **Muhammad Arshad:** Conceptualization (equal); resources (equal); validation (equal). **Sadia Kharal:** Resources (equal); validation (equal); visualization (equal). **Isam A. Mohamed Ahmed:** Validation (equal); visualization (equal); writing – review and editing (equal). **Shakila Khalid:** Data curation (equal); formal analysis (equal); visualization (equal).

## FUNDING INFORMATION

The research was funded by King Saud University, Riyadh, Saudi Arabia, through Researchers Supporting Project number (RSPD2024R1074).

## CONFLICT OF INTEREST STATEMENT

The authors have no conflict of interest to declare.

## Data Availability

The data supporting the conclusions of this article are included in the manuscript.

## References

[fsn34123-bib-0001] Abdulgafor, A. B. , Owain, M. S. , Hasan, M. S. , Hussein, M. A. , Aboud, Q. M. , & Al‐Zobaie, A. J. (2018). Liver, kidney function tests and oxidative damage during and after treatment of *salmonella typhimurium* infection in experimental local rabbits. International Journal of Pharmaceutical Quality Assurance, 9(4), 377–380.

[fsn34123-bib-0002] Alam, S. , Rashid, M. A. , Sarker, M. , Rahman, M. , Emon, N. U. , Arman, M. , Mohammed, I. N. , & Haque, M. R. (2021). Antidiarrheal, antimicrobial and antioxidant potentials of methanol extract of *Colocasia gigantea* hook leaves: Evidenced from *in vivo* and *in vitro* studies along with computer‐aided approaches. BMC Complementary Medicine and Therapies, 21(1), 1–12. 33845836 10.1186/s12906-021-03290-6 PMC8042880

[fsn34123-bib-0003] Albayrak, H. , & Kabu, M. (2016). Determining serum haptoglobin and cytokine concentrations in diarrheic calves. Firat University Veterinary Journal of Health Sciences, 30, 113–117.

[fsn34123-bib-0004] Alemayehu, H. , Nedi, T. , & Engidawork, E. (2022). Antidiarrheal and antibacterial activities of *Calpurnia aurea*: Benth seed different extracts. Evidence‐based Complementary and Alternative Medicine, 2022, 1–10. 10.1155/2022/9582687 PMC9451978 36091586

[fsn34123-bib-0006] Anouar, B. S. , Brahmi, D. , Ilhem, R. , Amani, S. , Sana, N. , Nacim, Z. , & Lazhar, Z. (2017). Phytochemical, antioxidant and protective effect of cactus cladodes extract against lithium induced liver injury in rats. Pharmaceutical Biology, 55(1), 516–525. 27951739 10.1080/13880209.2016.1255976 PMC6130665

[fsn34123-bib-0007] Baser, D. F. , & Civelek, T. (2013). Correlations between venous acid‐base status and renal function in neonatal calves with acute diarrhea. Kocatepe Veterinary Journal, 6, 25–31.

[fsn34123-bib-0008] Benayad, Z. , Martinez‐Villaluenga, C. , Frias, J. , Gomez‐Cordoves, C. , & Es‐Safi, N. E. (2014). Phenolic composition, antioxidant and anti‐inflammatory activities of extracts from Moroccan *Opuntia ficus‐indica* flowers obtained by different extraction methods. Industrial Crops and Products, 62, 412–420.

[fsn34123-bib-0009] Bozukluhan, K. , Merhan, O. , Gokce, H. I. , Deveci, H. A. , Gokce, G. , Ogun, M. , & Marasli, S. (2017). Alterations in lipid profile in neonatal calves affected by diarrhea. Veterinary World, 10(7), 786–789. 28831223 10.14202/vetworld.2017.786-789 PMC5553148

[fsn34123-bib-0010] Campos, S. F. , Chagas, A. C. , Costa, A. B. , França, R. E. , & Jansen, A. K. (2010). Fatoresassociadosaodesenvolvimento de úlceras de pressão: o impacto da nutrição. Annual Review of Nutrition, 23(5), 703–714.

[fsn34123-bib-0011] Chegaing, S. P. F. , Mefokou, D. Y. , Tangue, B. T. , Sokoudjou, J. B. , Menoudji, S. T. , Kamsu, G. T. , & Gatsing, D. (2020). Contribution to the ethnobotanical inventory of medicinal plants used for the treatment of typhoid fever in Adamaoua region, Cameroon. International Journal of Biological and Chemical Sciences, 14(9), 3078–3096.

[fsn34123-bib-0012] Del, R. J. C. , & Gutiérrez, A. (2008). Rapid assessment of the lignin composition of lignocellulosic materials commonly used for paper pulp manufacturing by analytical pyrolysis. Proceedings of the 18th International Symposium on Analytical and Applied Pyrolysis; Lanzarote, Spain, May 18−23, p. 99.

[fsn34123-bib-0013] Dey, A. , & De, J. N. (2012). Ethnobotanical survey of Purulia district, West Bengal, India for medicinal plants used against gastrointestinal disorders. Journal of Ethnopharmacology, 143(1), 68–80. 22721882 10.1016/j.jep.2012.05.064

[fsn34123-bib-0014] Donald, S. T. , Donatien, G. , Siméon, P. C. F. , Charles, F. , Fabrice, K. , & Merline, N. D. (2015). *In vivo* anti‐salmonella activity of aqueous extract of *Euphorbia prostrata* Aiton (Euphorbiaceae) and its toxicological evaluation. Asian Pacific Journal of Tropical Biomedicine, 5(4), 310–318.

[fsn34123-bib-0015] Eman, Y. A. , Marwa, I. E. , Hala, M. E. H. , & Essam, A. S. (2022). An overview and update on the chemical composition and potential health benefits of *Opuntia ficus‐indica* (L.) Miller. Journal of Food Biochemistry, 46(11), e14310. 35780308 10.1111/jfbc.14310

[fsn34123-bib-0018] Feingold, K. R. , Pollock, A. S. , Moser, A. H. , Shigenaga, J. K. , & Grunfeld, C. (1995). Discordant regulation of proteins of cholesterol metabolism during the acute phase response. Journal of Lipid Research, 36, 1474–1482. 7595071

[fsn34123-bib-0019] Feugang, J. M. , Konarski, P. , Zou, D. , Stintzing, F. C. , & Zou, C. (2020). Nutritional and medicinal use of cactus pear (*Opuntia* spp.) cladodes and fruits. Frontiers in Bioscience, 11(1), 2574–2589. 10.2741/1992 16720335

[fsn34123-bib-0020] Friedewald, W. T. , Levy, R. I. , & Frederickson, D. S. (1972). Estimation of concentration of the low‐density lipoprotein cholesterol in plasma, without use of preparative ultracentrifuge. Clinical Chemistry, 18, 449–502. 4337382

[fsn34123-bib-0024] Keast, D. H. , & Fraser, C. (2004). Treatment of chronic skin ulcers in individuals with anemia of chronic disease using recombinant human erythropoietin (EPO): A review of four cases. Ostomy/Wound Management, 50(10), 64–70. 15509883

[fsn34123-bib-0025] Kim, T. M. , Kim, K. H. , Jo, J. H. , Park, J. , Kwon, Y. S. , & Yang, J. H. (2020). Hepatoprotective effect of a novel lactic acid‐fermented garlic extract functional food product against acute liver injury. Food Science & Nutrition, 8(2), 1012–1019. 32148809 10.1002/fsn3.1385 PMC7020270

[fsn34123-bib-0026] Kouitcheu, M. L. B. , Kuiate, J. R. , & Oyono, E. J. L. (2011). Screening of some plants used in the Cameroonian folk medicine for the treatment of infectious diseases. International Journal of Biology, 3, 13–21.

[fsn34123-bib-0027] Lee, J. A. , Jung, B. G. , Kim, T. H. , Lee, S. G. , Park, Y. S. , & Lee, B. J. (2013). Dietary feeding of *Opuntia humifusa* inhibits UVB radiation‐induced carcinogenesis by reducing inflammation and proliferation in hairless mouse model. Photochemistry and Photobiology, 89(5), 1208–1215. 23789636 10.1111/php.12113

[fsn34123-bib-0028] Legba, B. , Dougnon, V. , Chabi, Y. , Gbaguidi, C. , Aniambossou, A. , Deguenon, E. , Dougnon, J. , & Baba‐Moussa, L. (2020). Evaluation of in‐vivo anti‐salmonella activity of *Uvaria chamae*, *Lantana camara* and *Phyllantus amarus* used in Benin, West Africa. BMC Veterinary Research, 16(1), 1–18. 32041607 10.1186/s12917-020-2266-1 PMC7011350

[fsn34123-bib-0029] Levy, A. , Fraser, D. , Rosen, S. D. , Dagan, R. , Deckelbaum, R. J. , & Coles, C. (2005). Anemia as a risk factor for infectious diseases in infants and toddlers: Results from a prospective study. European Journal of Epidemiology, 20, 277–284. 15921046 10.1007/s10654-004-6515-6

[fsn34123-bib-0030] Lutterodt, G. D. , Ismail, I. , Basheer, R. H. , & Mohd, B. H. (1999). Antimicrobial effects of *Psidium guajava* extract as one mechanism of its antidiarrhoeal action. Malaysian Journal of Medical Sciences, 6(2), 17–20. PMC3329747 22589684

[fsn34123-bib-0031] Ly, H. , Francone, O. L. , Fielding, C. J. , Shigenaga, J. K. , Moser, A. H. , Grunfeld, C. , & Feingold, K. R. (1995). Endotoxin and TNF lead to reduced plasma LCAT activity and decreased hepatic LCAT mRNA levels in Syrian hamsters. Journal of Lipid Research, 36, 1254–1263. 7666003

[fsn34123-bib-0032] Merhan, O. , Bozukluhan, K. , Gokce, G. , & Yilmaz, O. (2016). Investigation on the levels of haptoglobin, ceruloplasmin and some biochemical parameters levels in calves with diarrhea. Firat University Veterinary Journal of Health Sciences, 30, 195–198.

[fsn34123-bib-0033] Minato, K. , Miyake, Y. , Fukumoto, S. , Yamamoto, K. , Kato, Y. , Shimomura, Y. , & Osawa, T. (2003). Lemon flavonoid, eriocitrin, suppresses exercise induced oxidative damage in rat liver. Life Sciences, 72, 1609–1616. 12551749 10.1016/s0024-3205(02)02443-8

[fsn34123-bib-0058] National Committee for Research Ethics in Science and Technology (NENT) . (2019). Ethical Guidelines for the Use of Animals in Research: Access. https://www.forskningsetikk.no/en/guidelines/science‐and‐technology/ethical‐guidelines‐for‐the‐use‐of‐animals‐in‐research/

[fsn34123-bib-0034] Nesreen, M. E. S. , Ashraf, I. N. , Zainab, A. R. , & Sahar, F. D. (2021). Prickly pear [*Opuntia ficus indica* (L.) mill] peels: Chemical composition, nutritional value and protective effect on liver and kidney functions and cholesterol in rats. Functional Plant Science and Biotechnology, 5(1), 30–35.

[fsn34123-bib-0036] Nguyen, X. M. A. , Bun, S. S. , Ollivier, E. , & Dang, T. P. T. (2019). Ethnobotanical study of medicinal plants used by K'Ho‐Cil people for treatment of diarrhea in lam Dong Province, *Vietnam* . Journal of Herbal Medicine, 2020(19), 100320.

[fsn34123-bib-0037] Notio, M. , Sakurna, S. , & Miyazaki, A. (2003). Intravenous injections of rabbit apo‐lipoprotein habits progression of atherosclerosis in cholesterol fed rabbits. Arteriosclerosis, Thrombosis, and Vascular Biology, 15, 1882–1885. 10.1161/01.atv.15.11.1882 7583568

[fsn34123-bib-0038] Olatoye, K. K. , & Arueya, G. L. (2018). Toxicological parameters of albino rats fed with extruded snacks from aerial yam (*Dioscoria bulbifera*) and African breadfruit seed (*Treculia africana*). Food Science & Nutrition, 6(1), 94–100. 29387366 10.1002/fsn3.533 PMC5778208

[fsn34123-bib-0039] Omage, S. O. , Orhue, N. E. , & Omage, K. (2019). Evaluation of the phytochemical content, in vitro antioxidant capacity, biochemical and histological effects of *Dennettia tripetala* fruits in healthy rats. Food Science & Nutrition, 7(1), 65–75. 30680160 10.1002/fsn3.792 PMC6341132

[fsn34123-bib-0040] Osorio, E. O. , Álvarez, V. B. , Dorantes, A. L. , & Giusti, M. M. (2011). Phenolics, betacyanins and antioxidant activity in *Opuntia joconostle* fruits. Food Research International, 44(7), 2160–2168.

[fsn34123-bib-0041] Ozgun‐Acar, O. , Celik‐Turgut, G. , Guner, H. , Sezer, S. , & Sen, A. (2022). Biochemical, pharmacological, and toxicological attributes of caper (*Capparis ovata*) flowering buds and berries pickles. Food Science & Nutrition, 10(12), 4189–4200. 36514771 10.1002/fsn3.3012 PMC9731540

[fsn34123-bib-0042] Paiz, R. C. , Juárez, F. B. I. , Cecilia, J. R. A. R. N. , Ortega, C. , Aguuml, J. A. R. , Chávez, E. G. , & Fuentes, G. Á. (2010). Glucose‐lowering effect of xoconostle (*Opuntia joconostle* a. web., Cactaceae) in diabetic rats. Journal of Medicinal Plant Research, 4(22), 2326–2333.

[fsn34123-bib-0043] Panritdum, P. , Muangnoi, C. , Tuntipopipat, S. , Charoenkiatkul, S. , & Sukprasansap, M. (2024). *Cleistocalyx nervosum* var. paniala berry extract and cyanidin‐3‐glucoside inhibit hepatotoxicity and apoptosis. Food Science & Nutrition. 10.1002/fsn3.3975 PMC11016384 38628219

[fsn34123-bib-0044] Park, C. Y. , Choi, E. , Yang, H. J. , Ho, S. H. , Park, S. J. , Park, K. M. , & Kim, S. H. (2020). Efficacy of *Artemisia annua* L. extract for recovery of acute liver failure. Food Science & Nutrition, 8(7), 3738–3749. 32724636 10.1002/fsn3.1662 PMC7382175

[fsn34123-bib-0046] Pierre, D. , Etienne, C. , & Hans, P. (2017). Serum creatinine: Not so simple. Nephron, 136, 302–308. 28441651 10.1159/000469669

[fsn34123-bib-0047] Rahmani, S. , Naraki, K. , Roohbakhsh, A. , Hayes, A. W. , & Karimi, G. (2023). The protective effects of rutin on the liver, kidneys, and heart by counteracting organ toxicity caused by synthetic and natural compounds. Food Science & Nutrition, 11(1), 39–56. 36655104 10.1002/fsn3.3041 PMC9834893

[fsn34123-bib-0048] Rasool, N. , Omer, M. O. , Javeed, A. , Nawaz, M. , Imran, M. , Hussain, M. , Mushtaq, Z. , & Al Jbawi, E. (2023). Pharmacological effect of *Argyrolobium roseum* (Camb.) Jaub & Spach extracts against lead‐induced toxicity in rats. Food Science & Nutrition, 11(10), 6312–6323. 37823099 10.1002/fsn3.3570 PMC10563752

[fsn34123-bib-0049] Razavi, S. M. , Nazifi, S. , Rakhshandehroo, E. , Firoozi, P. , & Farsandaj, M. (2012). Erythrocyte antioxidant systems, lipid peroxidation and circulating lipid profiles in cattle naturally infected with *Theileria annulate* . Revue de Médecine Vétérinaire, 163, 18–24.

[fsn34123-bib-0050] Rishi, P. , Mavi, S. K. , Bharrhan, S. , Shukla, G. , & Tewari, R. (2009). Protective efficacy of probiotic alone or in conjunction with a prebiotic in salmonella‐induced liver damage. FEMS Microbiology Ecology, 69(2), 222–230. 19496820 10.1111/j.1574-6941.2009.00703.x

[fsn34123-bib-0051] Samadi‐Noshahr, Z. , Hadjzadeh, M. A. R. , Moradi‐Marjaneh, R. , & Khajavi‐Rad, A. (2021). The hepatoprotective effects of fennel seeds extract and trans‐Anethole in streptozotocin‐induced liver injury in rats. Food Science & Nutrition, 9(2), 1121–1131. 33598196 10.1002/fsn3.2090 PMC7866591

[fsn34123-bib-0052] Schiller, L. R. , Pardi, D. S. , & Sellin, J. H. (2017). Chronic diarrhea: Diagnosis and management. Clinical Gastroenterology and Hepatology, 15(2), 182–193. 27496381 10.1016/j.cgh.2016.07.028

[fsn34123-bib-0053] Sher, Y. , & Hung, M. (2009). Blood AST, ALT and UREA/BUN level analysis. Bio‐Protocol, 3(19), 931.

[fsn34123-bib-0056] Wang, G. K. , Zhang, N. , Wang, Y. , Liu, J. S. , Wang, G. , Zhou, Z. Y. , Lu, C. C. , & Yang, J. S. (2019). The hepatoprotective activities of *Kalimeris indica* ethanol extract against liver injury in vivo. Food Science & Nutrition, 7(11), 3797–3807. 31763029 10.1002/fsn3.1241 PMC6848823

